# Hsp90 inhibitor 17-allylamino-17-demethoxygeldanamycin inhibits the proliferation of ARPE-19 cells

**DOI:** 10.1186/1423-0127-17-30

**Published:** 2010-04-23

**Authors:** Jia-Qi Yao, Qing-Huai Liu, Xi Chen, Qin Yang, Zhi-Yang Xu, Fan Hu, Lin Wang, Jian-Min Li

**Affiliations:** 1 Department of Ophthalmology, the First Affiliated Hospital with Nanjing Medical University, 300 Guangzhou Road, Nanjing, Jiangsu, 210029 PR China; 2 Lab of Reproductive Medicine, Department of Cell Biology and Medical Genetics, Nanjing Medical University, 140 Hanzhong Road, Nanjing, Jiangsu, 210029 PR China

## Abstract

**Background:**

The antiproliferative effect of the Hsp90 inhibitor 17-AAG (17-allylamino-17-demethoxygeldanamycin) on human retinal pigment epithelial cells is investigated.

**Methods:**

MTT and flow cytometry were used to study the antiproliferative effects of the 17-AAG treatment of ARPE-19 cells. 2D gel electrophoresis (2-DE) and mass spectrometry were applied to detect the altered expression of proteins, which was verified by real-time PCR. Gene Ontology analysis and Ingenuity Pathway Analysis (IPA) were utilized to analyze the signaling pathways, cellular location, function, and network connections of the identified proteins. And SOD assay was employed to confirm the analysis.

**Results:**

17-AAG suppressed the proliferation of ARPE-19 cells by inducing cell cycle arrest and apoptosis. Proteomic analysis revealed that the expression of 94 proteins was altered by a factor of more than 1.5 following exposure to 17-AAG. Of these 94, 87 proteins were identified. Real-time PCR results indicated that Hsp90 and Hsp70, which were not identified by proteomic analysis, were both upregulated upon 17-AAG treatment. IPA revealed that most of the proteins have functions that are related to oxidative stress, as verified by SOD assay, while canonical pathway analysis revealed glycolysis/gluconeogenesis.

**Conclusions:**

17-AAG suppressed the proliferation of ARPE-19 cells by inducing cell cycle arrest and apoptosis, and possibly by oxidative stress.

## Background

The pathogenesis of some eye diseases involves the proliferation of retinal pigment epithelial (RPE) cells. For example, proliferative vitreoretinopathy (PVR) is responsible for most failures of the repair of retinal detachment following retinal reattachment surgery or severe ocular trauma, potentially resulting in significant loss of vision [1]. Although vitrectomy is helpful in reducing traction on the retina, removing vitreous opacities, and providing access to the vitreous cavity and retina in many surgical procedures, the rate of recurrence that ultimately leads to vision loss is high. Additional methods are required to prevent the formation of epiretinal membranes and contraction following vitreous surgery. Early PVR is characterized by dedifferentiation, migration, and proliferation of different cells, including fibroblasts, glial and RPE cells at the vitreoretinal interface, leading to the formation of contractile fibrocellular membranes on the surface of the retina. The proliferation and migration of RPE cells is an important step in PVR. RPE cells detach from the monolayer, migrate into the vitreous cavity and settle on the retina, forming a periretinal membrane. Since RPE cells are the main cells that are involved in the pathogenesis of PVR, research into therapies for PVR tend to focus on RPE cells. The inhibition of either RPE migration or RPE proliferation is a reasonable target for the development of drugs for treating PVR, so many chemotherapeutic agents have been used to inhibit the proliferation or migration of RPE cells to ameliorate PVR [[1,2] and [3]]. Most investigations, however, have not been successful.

Numerous antiproliferative and anti-inflammatory agents, such as retinoic acid, corticosteroid, and 5-fluorouracil (5-FU) have been tested to evaluate their potential to prevent the proliferation and migration of RPE cells, and thereby reduce traction retinal detachment in experimental models of PVR. Most of these drugs (many of which are antitumor drugs) have limited clinical application because of their toxicity [1,2]. Recently, a new antitumor drug, 17-AAG (17-allylamino-17-demethoxy-geldanamycin), was developed. Results from adult phase I studies reveal that 17-AAG can be administered without excessive toxicity, down-regulating several biomarkers, such as Raf-1, CDK4, Akt and others [4,5]. Hence, we hypothesize that this new drug may inhibit the proliferation of RPE cells, and may be effective in the treatment of PVR.

17-AAG, a derivative of the ansamycin geldanamycin, is less hepatotoxic than geldanamycin. Both 17-AAG and geldanamycin induce cell cycle arrest and apoptosis in various tumors. 17-AAG, which interferes with the function of Hsp90 (heat shock protein 90), is currently undergoing phase II/III clinical trials as the first Hsp90 inhibitor to treat tumors. Hsp90 is a chaperone protein that participates in the regulation of the intracellular disposition of diverse cellular proteins, including the components of signaling pathways that are exploited by cancer cells for survival and proliferation [6]. The inhibition of Hsp90 disrupts the folding of these proteins, increasing their susceptibility to ubiquitinization and proteasomal degradation. The exposure of cells to 17-AAG or geldanamycin down-regulates the expression of diverse signal transduction and cell cycle regulatory proteins, including Raf-1, cyclin D, and Akt, among others [7,8]. The Hsp90 inhibitor geldanamycin inhibits RPE cell proliferation [9]. However, the full range of effects of Hsp90 inhibition in RPE cells are unknown.

Proteomic analysis is a valuable method for elucidating the molecular nature of Hsp90 inhibition. Many researchers have used it to study cellular protein changes following exposure of cancer cell lines to 17-AAG or geldanamycin or other Hsp90 inhibitors [[6,10] and [11]]. This work tested the antiproliferative effects of 17-AAG on the human RPE cell line ARPE-19, as well as the change in protein expression. Real-time PCR was used to verify the change in expression of selected genes that were identified by proteomic analysis, as well as changes in the expression of Hsp90 and Hsp70, which were not thus identified. Gene Ontology analysis and Ingenuity Pathways Analysis (IPA) was applied to analyze the molecular functions, signal pathways, cellular location and network connections of the proteins, as well as their possible involvement in disease processes.

## Methods

### Human RPE cell culture and reagents

The human RPE cell line ARPE-19 (American Type Culture Collection, Manassas, VA) was cultured in DMEM/F12 (Hyclone, Logan, UT) supplemented with 10% fetal calf serum (FCS; Gibco, Grand Island, NY), 100 μg/ml penicillin and 100 μg/ml streptomycin (both from Gibco) and maintained at 37°C and 5% CO2 in a humid atmosphere. In all experiments, dimethyl sulfoxide (DMSO; Sigma-Aldrich, St. Louis, MO) was added as a negative control. 17-AAG (Sigma-Aldrich) was dissolved in DMSO, which was then stored at -20°C.

### Cell viability analysis

ARPE-19 cells were seeded in 96-well plates, in 100 μl of growth medium. Following an overnight attachment period, the cells were exposed to various concentrations of 17-AAG (0, 0.05, 0.1, 0.5, 1, 5 and 10 μM). All studies were performed at least three times independently. At the end of the treatment period (24, 48 or 72 h), 20 μl of a 5 mg/ml stock solution of 3-(4, 5-dimethylthiazol-2-yl)-2, 5-diphenyltetrazolium bromide (MTT; Sigma-Aldrich) was added to each well. Four hours later, 150 μl DMSO was added to dissolve the dark blue formazan crystals that were formed by the living cells. The absorbance of each well at 490 nm (A_490_) was determined using a Bio-Rad model 450 microplate reader (Bio-Rad Laboratories, Richmond, CA). The background A_490 _of the wells that did not contain cells was subtracted before the percentage of viable cells was calculated [(A_490 _of 17-AAG treated sample/A_490 _untreated cells) × 100%].

### Annexin-V and Propidium Iodide (PI) staining

To identify cells in the early and late stages of apoptosis, the Annexin V-FITC Apoptosis Detection Kit was applied (BD PharMingen, San Diego, CA). Briefly, ARPE-19 cells were cultured with 3 μM 17-AAG for 24, 48 or 72 h, trypsinized and washed twice with cold PBS (phosphate-buffered saline), and then resuspended in 1 × binding buffer (BD PharMingen) at a concentration of 1 × 10^6 ^cells/ml. One hundred μl of the cell suspension was transferred to a 5 ml polypropylene tube, and 5 μl each of PI (50 μg/ml stock) and annexin V-FITC were added simultaneously. The cells were gently mixed and incubated at room temperature in the dark for 15 min. Three hundred μl of 1 × binding buffer was added to each tube, and the cells were analyzed immediately by flow cytometry (FACScan, Becton Dickinson, Franklin Lakes, NJ). The morphology of ARPE-19 cells that were treated with DMSO or various concentrations of 17-AAG (0.5 μM, 3 μM, 10 μM) for 24 h was recorded by visualizing the cells under an Olympus U-LH100HG light microscope (Olympus, Tokyo, Japan).

### Cell cycle analysis by flow cytometry

ARPE-19 cells were cultured with 3 μM 17-AAG for 24, 48 or 72 h, and then collected, washed twice with ice-cold 1× PBS, and fixed with 70% ethanol overnight at -20°C. Cell pellets were washed twice with ice-cold PBS, resuspended in PBS, and stained with PI that contained 100 μg/ml RNAse (Sigma-Aldrich). Stained cells were maintained on ice and protected from light. They were then analyzed on the FACScan flow cytometer, as described above. Data were analyzed using CellQuest Software (Becton Dickinson, Mountain View, CA).

### Isolation of cellular proteins

To prepare protein lysates from ARPE-19 cells preparations, the cells were incubated with 3 μM 17-AAG or DMSO for 16 h. Each treatment was performed in triplicate. After the cells were trypsinized and washed twice with PBS, 200 μl lysis buffer [7 M urea, 2 M thiourea, 4% (w/v) CHAPS, 1% (w/v) dithiothreitol (DTT), 1% Protease Inhibitor Cocktail (v/v), and 2% (v/v) IPG buffer (pH 3-10)] were added at 11,000 IU/min on ice (in ten bursts of 10 s each, separated by short pauses). Suspensions then were stored at 4°C for 1 h, before undergoing centrifugation at 40,000 × g for 60 min at 4°C. The supernatants were aliquoted and stored at -70°C. The protein concentration of each sample was measured by the Bradford method [12].

### Two-Dimensional Electrophoresis (2-DE)

Isoelectric focusing (IEF) was conducted using an Ettan IPGphor II (Amersham Bioscience, Uppsala, Sweden) with 24 cm immobilized pH gradient strips (pH 3-10; Amersham Bioscience). Samples that contained 300 μg of protein were mixed with rehydration solution [8 M urea, 2% CHAPS, 20 mM DTT, 0.5% (v/v) IPG buffer (pH 3-10), and 0.001% bromophenol blue]. The linear ramping mode of the IEF voltage was used as follows; 30 V for 6 h, 60 V for 6 h, 500 V for 1 h, 1000 V for 1 h, and 3000 V for 1 h, followed by 8000 V for approximately 7.5 h, to achieve 64 kVh at 20°C. Strips were then equilibrated at room temperature for 15 min in 10 ml equilibration solution [6 M urea, 50 mM Tris-HCl (pH8.8), 30% (v/v) glycerol, 2% sodium dodecyl sulfate (SDS), 1% (w/v) DTT] and incubated for another 15 min in an equilibration solution that was the same as previously used, except with DTT replaced by 2.5% (w/v) iodoacetamide]. Second-dimension electrophoresis was carried out on 12.5% SDS gels in the Ettan DALTsix at 5 W per gel for the first 30 min, followed by 12 W per gel for 6-7 h until the bromophenol blue line reached the bottom of the gels. Gels (three of each) were then silver-stained to visualize the in-gel proteins, following published methods described elsewhere [12].

Silver-stained gels were scanned using an Atrix scan 1010 plus (Microtek, Taiwan, China), and the resulting images were analyzed using the ImageMaster 2D Platinum software (Amersham Bioscience) for spot detection, quantification, and comparative and statistical analyses. The mean and SD normalized volume of each protein spot were calculated, and statistical comparisons between the intensity of the control and the 17-AAG treated spots were conducted using Student't-test (with p < 0.05 considered significant) [13].

### In-Gel digestion and MALDI-TOF analysis

Detected spots and four control spots (with volumes of around 1 mm^3^) were excised from the silver-stained gels and the nonstained areas of the gels, respectively. For in-gel protein digestion, the gel-bound proteins were washed at room temperature with 50 mM acetonitrile (ACN)/NH_4_HCO_3 _1:1(v/v), once for 10 min and once for 30 min, dehydrated in 20 μl ACN for 20 min, and then dried in a vacuum centrifuge (Eppendorf AG, Hamburg, Germany) for 30 min at 30°C. Proteins were reduced by incubation in 50 μl 10 mM DTT/25 mM NH_4_HCO_3 _at 56°C for 1 h and then alkylated in 50 μl 55 mM iodoacetamide/25 mM NH_4_HCO_3 _for 45 min at room temperature in the dark. The liquid was discarded, and gel pieces were washed twice in 25 mM NH_4_HCO_3_, dehydrated in ACN, and dried in a vacuum centrifuge for 30 min at 30°C. Gel pieces were then rehydrated in 4 μl 25 mM NH_4_HCO_3 _that contained 40 ng trypsin and incubated at 4°C for 1 h. Excess liquid was discarded and gel plugs were incubated overnight at 37°C, with tubes inverted to keep the gel pieces wet to ensure sufficient enzymatic cleavage. Then, 8 μl of 5% (v/v) trifluoroacetic acid (TFA) was added and samples were incubated at 37°C for 1 h; supernatants were collected and the proteins were extracted twice by incubating the gel pieces in 8 μl of 2.5% TFA/50% ACN at 37°C for 1 h. Supernatants were mixed and completely dried in a vacuum centrifuge. The resulting peptides were maintained at 4°C until they were analyzed on a mass spectrometer.

For MALDI-TOF (matrix-assisted laser desorption ionization-time of flight) analysis, the dried peptides were dissolved in 2 μl 0.5% TFA. The matrix material was dissolved in TA solution to saturation (ACN: 0.1% TFA: acetone = 3:6:1). The matrix and the analyte solution were mixed in a ratio of 1:1, and 1 μl of the mixture was deposited onto the stainless steel sample target. The solvent was allowed to evaporate at room temperature. MALDI-TOF analysis of trypsin digests was performed using a Bruker Biflex IV MALDI-TOF-MS (Bruker Daltonics, Germany) that was equipped with an N2 laser (337 nm, 3 ns pulse length) in positive ion mode at an accelerating voltage of 19 kV. Peptide data were collected in the reflectron mode. Each spectrum was formed from around 200 laser shots. External calibration for peptide analysis was performed using peptide calibration standards [12].

### Database research

Data were screened against the SWISS-PROT database using the MASCOT search program http://www.matrixscience.com. Searches were performed using peptide mass accuracy tolerances of 0.3 Da or 200 ppm for external calibration. Peptides in the blank controls were excluded. One missed cleavage per peptide was allowed. The variable modification was considered to be carbamidomethyl (C). The requirements for positiviely identifying proteins were as follows: (1) at least three matching peptide masses; and (2) molecular weight and isoelectric points (pI) of identified proteins should be consistent with the values estimated from the image analysis.

### RNA extraction and cDNA synthesis

For real-time PCR measurements, the ARPE-19 cells were treated with either 3 μM 17-AAG for 16, 24 or 48 h, or 2, 5, or 10 μM 17-AAG for 24 h. Total RNA was extracted from each sample using TRIzol (Invitrogen, Carlsbad, CA) following the manufacturer's instructions. The RNA concentration was determined by measuring the absorbance at 260 nm using a spectrophotometer. The extracted total RNA was treated with RNase-free DNase I to eliminate potential contamination by genomic DNA. Around 1 μg of total RNA from each sample was used in the synthesis of the first strand of cDNA. The synthesis of the first-strand cDNA was primed using oligo (dT) from the SuperScript III First-Strand synthesis kit (Invitrogen). The synthesized cDNA was used as a template to estimate the quantity of gene transcription by real-time PCR.

### Real-time PCR

Real-time PCR was used to confirm the 2-DE results concerning changes to the transcription of proteins. Real-time PCR was carried out using an ABI PRISM1 7300 Sequence Detection System (Applied Biosystems, Foster City, CA) and a TaqMan PCR Master Mix (Applied Biosystems) with a final volume of 20 μl. β-actin was the internal control. The PCR amplification protocol was as follows; 50°C for 2 min and 95°C for 10 min, followed by 40 cycles of 95°C for 15 s and 60°C for 30 s. Table 1 presents the primers and probes of the internal control and target genes. Each sample, including the internal control, was tested in triplicate. Each sample that was treated with 17-AAG was compared with one treated with the same volume of DMSO using the 2^-ΔΔCT ^method [14].

**Table 1:** Primers and probes for real-time PCR.

Gene ID	Gene name	Sequence*	Amplicon size(bp)
NM_000925	PDHB	F: 5'-GAAACCATAGAAGCCAGTGTCA-3' R: 5'-TCTTTGCATAAGGCATAGGGA-3' P: 5'-TGTGGAAGGAGGCTGGCCACA-3'	181
NM_005809	PRDX2	F: 5'-AGGTGAAGCTGTCGGACTACA-3' R: 5'-TGCTGAACGCGATGATCTC-3' P:5'-CGTGGTCCTCTTTTTCTACCCTCTGGA-3'	99
NM_006406	PRDX4	F: 5'-CACTTCTACGCGGGTGGA-3' R: 5'-CGCTGGCTTGGAAATCTT-3' P: 5'-CGCCGACCACTCCCTGCACC-3'	102
NM_005345	HSPA1A	F: 5'-CTGCGACAGTCCACTACCTTTT-3' R: 5'-TCCCTGCTCTCTGTCGGC-3' P: 5'-CCAAGGCTTCCCAGAGCGAAC-3'	187
NM_002156	HSPD1	F: 5'-GGAGTGGCTGTGCTGAAGGT-3' R: 5'-GCATCGAAGGAGGGCACA-3' P: 5'-CTGCTGTTGAAGAAGGCATTGTTTTG-3'	144
NM_001017963	HSP90AA1	F: 5'-TTCAGACAGAGCCAAGGTGC-3' R: 5'-CAATGACATCAACTGGGCAAT-3' P: 5'-CCCAGACCCAAGACCAACCGATGG-3'	168
NM_007355	HSP90AB1	F: 5'-GGCAGTCAAGCACTTTTCTGTAG-3' R: 5'-GTCAACCACACCACGGATAAA-3' P: 5'-ATTGCTATTTATTCCTCGTCGGGCT-3'	199
NM_002046	GAPDH	F: 5-TGCACCACCAACTGCTTAGC-3' R: 5'-TCTTCTGGGTGGCAGTGATG-3' P: 5'-ATGGACTGTGGTCATGAGTCCTTCCA-3'	106
NM_001100	β-actin	F: 5'-GGCACCCAGCACAATGAA-3' R: 5'-GGAAGGTGGACAGCGAGG-3' P: 5'-CAAGATCATTGCTCCTCCTGAGCGC-3'	98

*F: forward; R: reverse; P: probe

### Analysis of molecular functions and pathway usingbioinformatics

Regulated proteins identified by 2-DE were analyzed further by Gene Ontology (GO) analysis and Ingenuity Pathway Analysis (IPA; Ingenuity Systems, Mountain View, CA;http://www.ingenuity.com). GO is a structured, controlled vocabulary that describes geneproducts in terms of their associated biological processes, cellular components and molecular functions in a manner that does not depend on species [15]. IPA was used as described by the manufacturer to model specific physiological processes that were influenced by exposure to 17-AAG. Each gene name was mapped to its corresponding gene object (node) in the Ingenuity Pathways Knowledge Base (IPKB). The magnitude and direction of changes to the gene objects were determined by forming a ratio between them, which reveals statistically significant changes caused by exposure to 17-AAG as well as their control levels before exposure. To obtain as complete a picture as possible, the ratios for the increased and decreased gene objects were combined into a single dataset. The IPA program treated these ratios as change factors, and gene objects whose changed by a factor of at least two were overlaid onto a global molecular network that was developed from information that was contained in the IPKB. Networks were then algorithmically generated based on their connectivity.

### SOD assay

The ARPE-19 cells were treated with 3 μM 17-AAG for 16, 24 or 48 h, and then SOD activity was determined using an SOD Assay Kit-WST (Dojindo Molecular Technologies, Inc. Japan) following the manufacturer's directions. Cells with added DMSO were used as controls. This kit supports highly sensitive SOD assay using a highly water-soluble tetrazolium salt, WST-1 [2-(4-iodophenyl)-3-(4-nitrophenyl)-5-(2, 4-disulfo-phenyl)-2H-tetrazolium, monosodium salt], which forms a water-soluble formazan dye upon reduction by a superoxide anion. A colorimetric assay is employed to measure the amount of formazan produced by the reaction between WST-1 and the superoxide anion (O_2_^-^); the rate of reduction with O_2_^- ^is linearly related to the xanthine oxidase activity which is inhibited by SOD. The absorbance was obtained using a microplate reader at 450 nm. SOD activity (inhibition rate %) = [(A_blank1 _- A_blank3_) - (A_sample _- A_blank2_)]/(A_blank1 _- A_blank3_) × 100. SOD activity per well was determined from the standard curve, and the amount of SOD per cell was calculated. Data were normalized to the control.

### Statistical analysis

All experiments were performed in triplicate and all data were presented as mean ± S.D. When applicable, differences between two groups were determined using the unpaired Student's t-test. For multigroup comparisons, ANOVA and then a Student-Newman-Keuls test were performed. P < 0.05 was regarded as statistically significant.

## Results

### 17-AAG inhibited proliferatin of RPE cells

**Figure 1:**
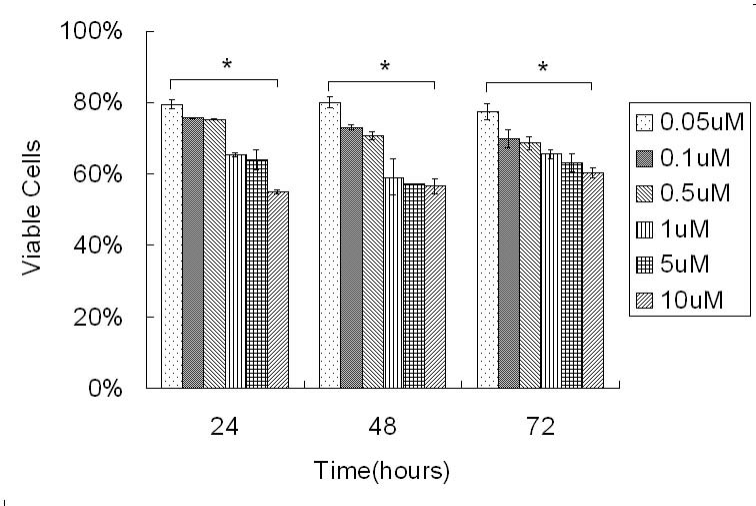
**17-AAG suppresses RPE cells proliferation**. ARPE-19 cells were treated with various concentrations of 17-AAG for 24, 48, or 72 h. Their viability was evaluated using the MTT assay, as described in the Methods section. Values are presented as means ± SD for triplicate cultures of each cell and are presented as the percentage of control cells that received medium only. 17-AAG reduces the viability of ARPE-19 cells in a dose-dependent manner (p < 0.05). No time-dependent decrease in viability occurs, regardless of the concentration of 17-AAG (p > 0.05). *, p < 0.05.

To study the biological effect of the inhibition of Hsp90 in ARPE-19 cells, the cells were incubated with increasing doses of 17-AAG (0 - 10 μM), and cell viability in each case was evaluated using the MTT proliferation assay. 17-AAG reduced cell viability in a dose-dependent manner (p < 0.05). No time-dependent decrease in viability occurred, regardless of the concentration of 17-AAG (p > 0.05). Viability at 72 h recovered slightly (Fig. 1). To prevent excessive apoptosis, a concentration of 3 μM was selected for the following experiments.

### 17-AAG induces apoptosis in ARPE-19 cells

**Figure 2:**
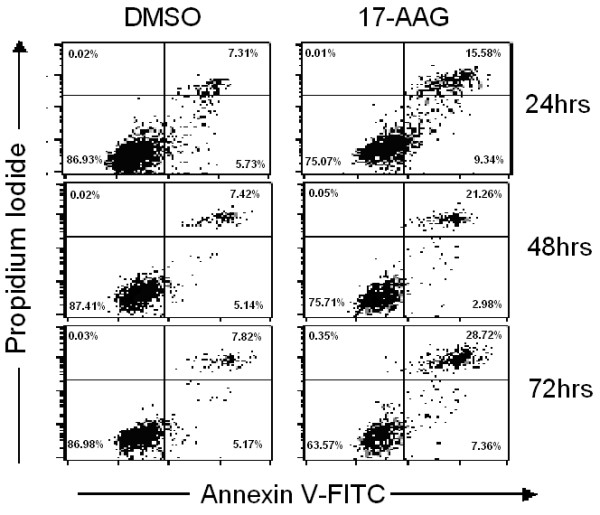
**17-AAG induces apoptosis in ARPE-19 cells**. Following incubation with 3 μM 17-AAG for 24, 48 or 72 h, apoptosis was assessed using a flow cytometer to detect cells that were stained with Annexin V or PI, as described in the Methods section. Exposure to 17-AAG causes a significant population of ARPE-19 cells to proceed to a later stage of apoptosis.

**Figure 3:**
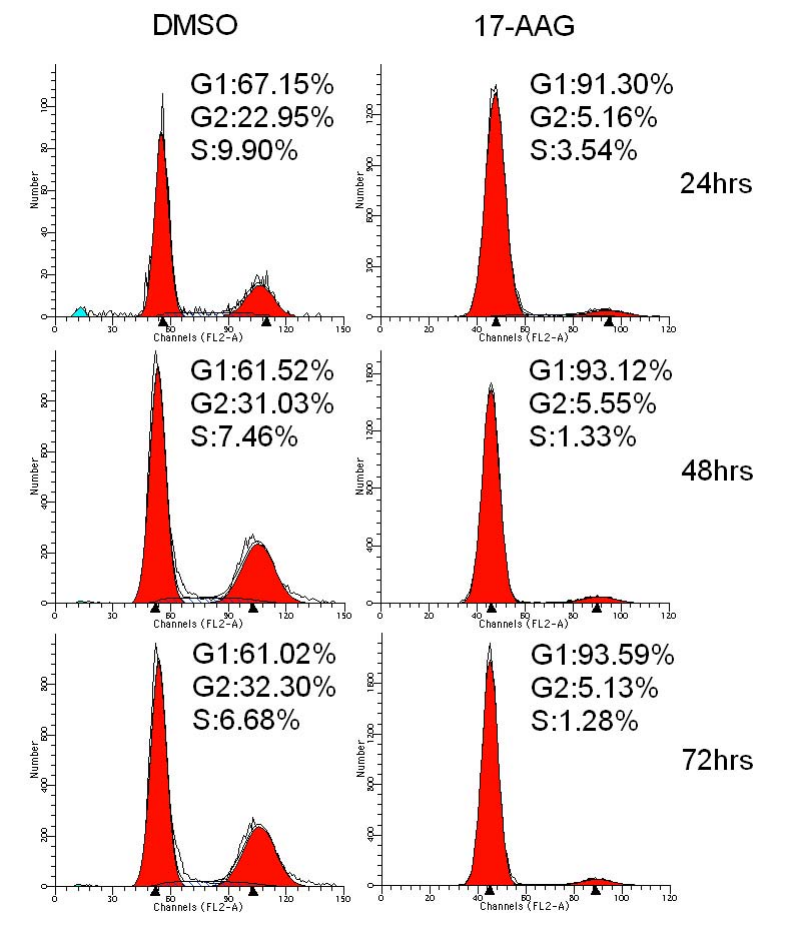
**The treatment of ARPE-19 cells with 17-AAG induces G1 cell cycle arrest**. After incubation with 3 μM 17-AAG for 24, 48 or 72 h, cell cycle progression was evaluated by flow cytometry, as described in the Methods section. 17-AAG induces G1 cell cycle arrest in ARPE-19 cells.

ARPE-19 cells were incubated with 3 μM 17-AAG or the same volume of DMSO for up to 72 h. The cells were then incubated with Annexin V-FITC in a buffer that contained PI and analyzed by flow cytometry. Most of the untreated cells were Annexin V-FITC and PI negative (Fig. 2, lower left quadrant), indicating that the cells were viable and did not undergo apoptosis. Following treatment with 17-AAG, a number of cells were Annexin V-FITC-positive but PI-negative (lower right quadrant), suggesting that they were in the early stages of apoptosis. Furthermore, a significant population of cells had progressed to a later stage of apoptosis and were stained by both PI and Annexin V. 17-AAG caused morphological changes when added to ARPE-19 cells, since numerous cells were detached and floated to the top of the culture medium, where they were shrunken and dispersed. The changes in cell morphology became increased as the concentration increased (data not shown). The induction of apoptosis was preceded by cell cycle arrest. The treatment of ARPE-19 cells with 17-AAG induced G1 cell cycle arrest as determined by comparison with control cells (Fig. 3).

### 2-DE and image analysis

**Figure 4:**
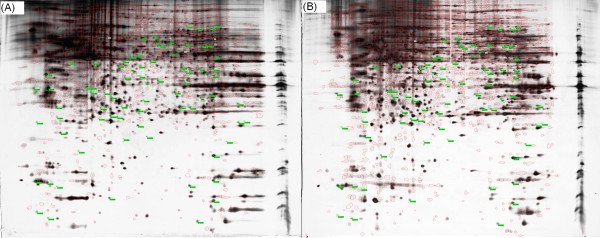
**2-DE reveals protein expression in untreated ARPE-19 cells or ARPE-19 cells exposed to 17-AAG**. Isoelectric focusing followed by second-dimension electrophoresis was carried out using protein lysates from ARPE-19 cells incubated with 3 μM 17-AAG or DMSO for 16 h. Gels (three of each) were then silver-stained and scanned, and the resulting images were analyzed using the ImageMaster 2D Platinum software for spot detection, quantification, and comparative and statistical analyses as described in the Methods section. 2-DE reveals protein expression in untreated ARPE-19 cells (A) or ARPE-19 cells exposed to 17-AAG (B). The green labels identify proteins whose expression is altered by a factor of greater than 1.5 in the treated cells.

The images of silver-stained gels were analyzed using ImageMaster 2D Platinum software. Figures 4A and 4B show images of control and those treated with 17-AAG. Based on a 1.5-fold change cutoff, the analysis identified 94 proteins (Fig. 4), while MALDI-TOF analysis identified 87 proteins whose expression was altered. Additional file 1 and 2 present the name, IPI Accession number, function, molecular weight and isoelectric point of each protein.

### Confirming altered expression with real-time PCR

**Figure 5:**
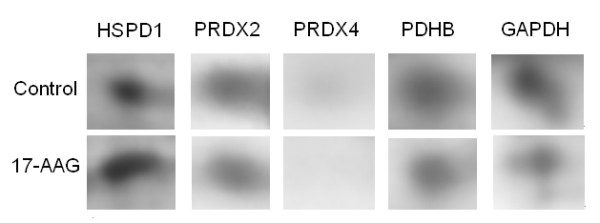
**Selected regions of 2D gels, showing changes in protein expression following treatment with 17-AAG**. Expression of protein in the 2D gels by HSPD1, PRDX2, PRDX4, PDHB and GAPDH following exposure to 3 μM 17-AAG for 16 h.

**Figure 6:**
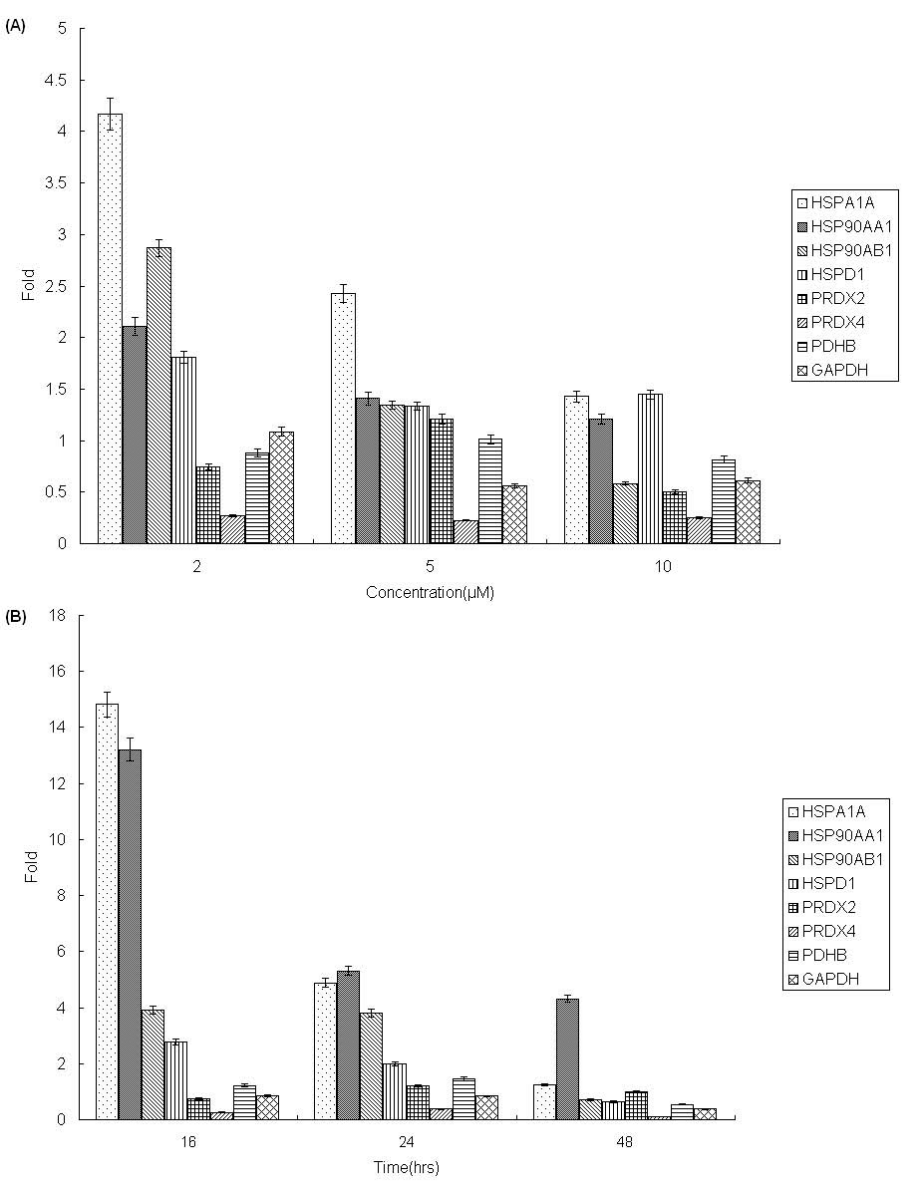
**Dose- and time-dependent changes in expression of selected genes upon exposure to 17-AAG**. After the ARPE-19 cells were treated with either 3 μM 17-AAG for 16, 24 or 48 h, or 2, 5, or 10 μM 17-AAG for 24 h, total RNA from each sample was extracted and reverse transcribed into cDNA. Changes in the expression of proteins were verified by real-time quantitative PCR. The dose (A) and time (B) -dependent changes were both evaluated as mean ± SD. The changes in selected genes are consistent with the results of proteomics. Another three unidentified proteins, Hsp70, Hsp90α and Hsp90β, were all upregulated by 17-AAG treatment, but their upregulation declined in a time and dose-dependent manner.

Real-time PCR, which rapidly and precisely quantifies gene expression levels, was utilized to verify the determined changes in protein expression. Five specific proteins that were differentially regulated by 17-AAG were selected, and their gene expression in response to the treatment of ARPE-19 cells by 17-AAG was quantified. Figure 5 displays the five selected regions of 2D gels, revealing individual protein expression changes upon exposure to 3 μM 17-AAG for 16 h. Proteomic analysis did not identify HSPA1A (Hsp70), HSP90AA1 (Hsp90α) or HAP90AB1 (Hsp90β), but they are commonly reported to change upon treatment with 17-AAG, and were also detected by real-time PCR. The real-time PCR verified the changes of the five selected proteins. Another three unidentified proteins were all upregulated by 17-AAG treatment, but their upregulation declined in a time and dose-dependent manner. Even Hsp90β was suppressed upon exposure to 10 μM 17-AAG for 24 h or 3 μM 17-AAG for 48 h. Notably, Hsp70 and Hsp90α were upregulated by a factor of more than 12 after 16 h of treatment with 3 μM 17-AAG (Figs. 6A and 6B).

### Bioinformatics analysis

**Figure 7:**
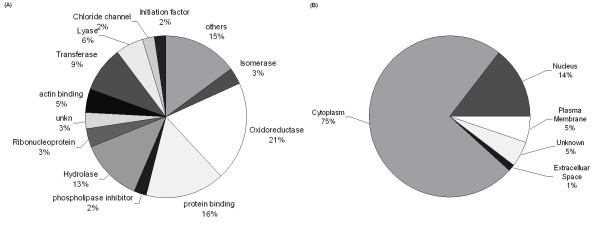
**Functional classification and subcellular localization of identified proteins based on bioinformatics**. Regulated proteins identified by 2-DE were analyzed further by Gene Ontology (GO) analysis and Ingenuity Pathway Analysis (IPA). GO analysis for functional classification of proteins identified by proteomics. Catalysis and protein binding are major functions of the identified proteins (A). IPA of subcellular localization of identified proteins, most of which are located in cytoplasm (B).

To identify major molecular functions that were affected by 17-AAG, the protein dataset was subjected to Gene Ontology (GO) analysis. Molecular functions that involved the most identified proteins were executed (Additional file 1 and 2 and Fig. 7A). Many proteins mapped to multiple functions. Catalytic activity accounted for 52% of the identified proteins, which were grouped into oxidoreductase (21%), hydrolase (13%), transferase (9%), lyase (6%), and isomerase (3%). Protein binding (16%) and actin binding (5%) were also major functions.

**Figure 8:**
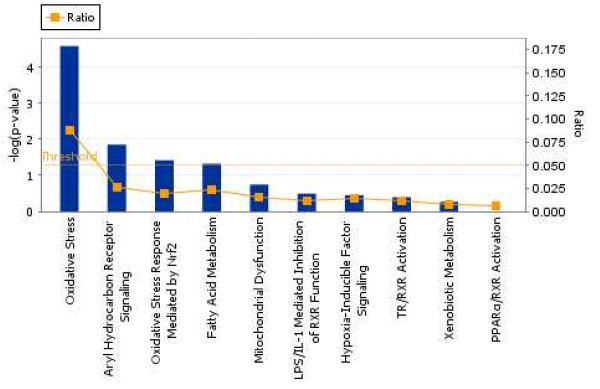
**Differentially expressed genes associated with a canonical pathway in the Ingenuity Pathway Analysis**. IPA was applied to model specific physiological processes that were affected by exposure to 17-AAG. Each gene name was mapped to its corresponding gene object (node) in the Ingenuity Pathways Knowledge Base (IPKB). Top function in the list is oxidative stress.

**Figure 9:**
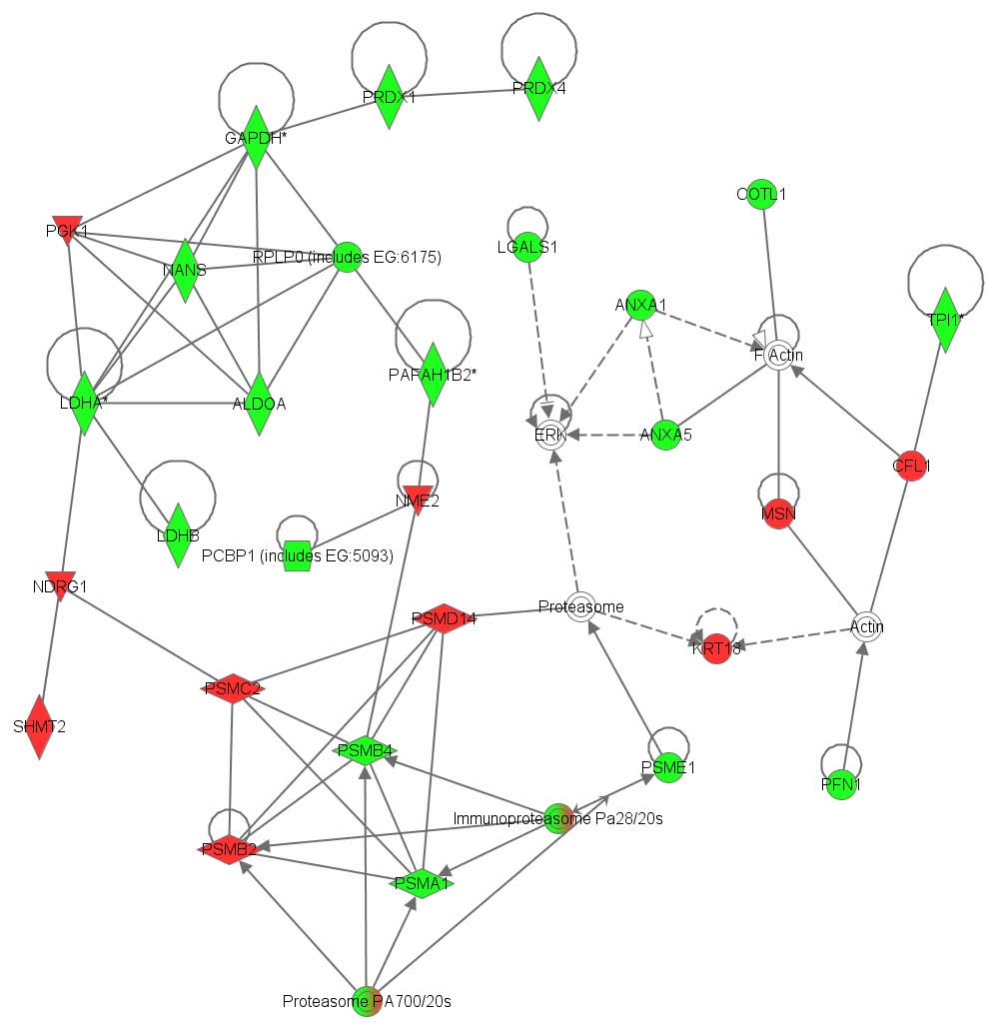
**Network that contains the largest number of differentially expressed proteins**. IPA was employed to identify biological networks that were affected by 17-AAG. The 87 unique IPI accession numbers were analyzed for network associations by the Ingenuity Knowledge base and six high ranking networks were identified. The top-ranked network comprises 35 proteins, including 29 that were identified by proteomics and six that were recognized as related to the network because of their reported interactions with the proteins that were identified by difference gel electrophoresis (DIGE). Shaded nodes are derived from dataset of proteins identified by 2D, and white nodes are inserted by the IPA program. Green represents a decrease, while red denotes an increase. Nodes are displayed using various shapes that represent the functional class of the gene product: concentric circles represent a group or complex; down-pointing triangles represent kinases; diamonds represents other enzymes; horizontal ovals represent transcription regulators; vertical ovals represent transmembrane receptors; vertical rectangles represent G-protein coupled receptors; horizontal rectangles represent ligand-dependent nuclear receptors, and circles represent other entities. Solid lines indicate direct interactions between nodes whereas dashed lines represent indirect interactions. Lines beginning and ending at a single node show self-regulation, while a line without an arrowhead represents binding. Arrowheads represent directionality of the relationship.

Ingenuity Pathways Analysis (IPA) was used to identify biological networks that were affected by 17-AAG. The 87 unique IPI accession numbers in Additional file 1 and 2 were analyzed for network associations of them using the Ingenuity Knowledge base, a curated database of publicly available pathways and relationships. Most identified proteins were located in the cytoplasm (Fig. 7B). The top function in the list was oxidative stress (Fig. 8). Six high ranking networks were identified. Figure 9 shows the top-ranked network. The significance of the association between the data set and the canonical pathway was measured in two ways: (1) a ratio of the number of genes from the data set that map to the pathway to the total number of genes that map to the canonical pathway is given; (2) Fischer's exact test was performed to calculate a p value for the probability that the association between the genes in the dataset and the canonical pathway is explained by chance alone. A pathway with a significance of less than 0.05 was regarded as significantly regulated.

### SOD assay

**Figure 10:**
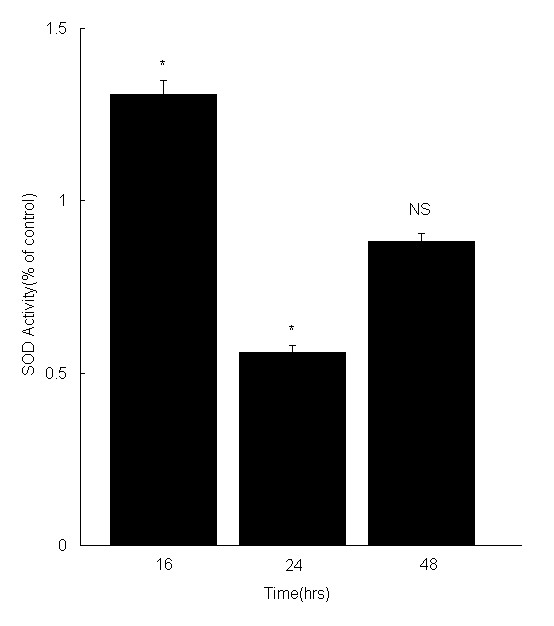
**Change in SOD activity after 3 μM 17-AAG treatment for 16 h, 24 h and 48 h**. The ARPE-19 cells were treated with 3 μM 17-AAG for 16, 24 or 48 h, and then SOD activity was determined by an SOD Assay Kit-WST. The SOD activity in RPE cells is upregulated in 16 h and downregulated in 24 h, as determine by comparison with control. However, the SOD activity in RPE cells is almost fully recovered in 48 h. Values are expressed as mean ± SD. *, p < 0.05, indicates a significant difference from control. NS, no significant difference from control.

To confirm the results of IPA, an SOD assay was utilized. Following incubation with 3 μM 17-AAG, the SOD activity in RPE cells was upregulated in 16 h and downregulated after 24 h, by comparison with a control, verifying the results of proteomics (Fig. 10).

## Discussion

Hsp90 is a molecular chaperone which facilitates the folding and stabilization of numerous protein kinases and intracellular signaling molecules. Hsp90 is a potential target for pharmacological inhibition in various cancer cells [16]. The inhibition of Hsp90 by 17-AAG or other Hsp90 inhibitors has a potent antitumor effect. The antiproliferative effects of 17-AAG and other Hsp90 inhibitors have been extensively studied in numerous cell lines. 17-AAG inhibits three signaling pathways - Raf/MEK/ERK, PI3K/AKT, and p38/MAPK - by inhibiting the activity of at least one member of the pathway [17,18]. All of these pathways may be involved in the proliferation of RPE cells, and inhibiting one of these pathways leads to the apoptosis of RPE cells [19,20]. 17-AAG inhibits the activity of cell cycle proteins, such as CDK4 and CDK6, and induces cell cycle arrest. 17-AAG also can induce apoptosis by activating caspases or in a caspase-independent manner [21]. 17-AAG also inhibits the function of some cytokines that are associated with PVR, such as EGF and PDGF, by inhibiting the interaction of Hsp90 with their receptors [22]. The authors' earlier investigation indicated that 17-AAG downregulates Akt-1, Raf-1 [8], and PDGFR [23] in ARPE-19 cells.

This work demonstrates that an Hsp90 inhibitor, 17-AAG, has a potent antiproliferative effect on ARPE-19 cells by inducing cell cycle arrest and apoptosis. MTT proliferation assay revealed the absence of a time-dependent decrease in viability, but flow cytometry indicated a time-dependent decline in apoptosis in the cells that were treated with 17-AAG. Because of the difference between the MTT analysis and flow cytometry, and the difference between the concentrations of 17-AAG used in the two methods herein, this discrepancy may be acceptable.

To investigate the mechanism of the antiproliferation effect of 17-AAG on ARPE-19 cells, changes in protein expression caused by treatment with 17-AAG were examined by 2-DE and MALDI-TOF. The 16 h time point was chosen because most of the proteins in other cell lines changed within 16 hours of treatment with 17-AAG [24]. Proteomics analysis (2-D electrophoresis and MALDI-TOF mass spectrometry) combined with bioinformatics analysis also revealed that catalytic activity accounted for 52% of the identified proteins and 17-AAG may inhibit the proliferation of ARPE-19 cells and induce aopotosis in part by inducing oxidative stress.

The proteins that were downregulated in RPE by 17-AAG included several glycometabolism-associated enzymes, including PDHB (pyruvate dehydrogenase), LDHB (L-lactate dehydrogenase B chain), TPI1 (isoform 1 of triosephosphate isomerase) and ALDOA (fructose-bisphosphate aldolase A). A canonical pathways analysis of IPA also highlighted one major pathway, glycolysis/gluconeogenesis. Some researchers have reported that the inhibition of Hsp90 influences metabolic pathways and a large-scale Hsp90-interactomic survey in yeast has revealed a huge proportion of metabolic enzymes as possible Hsp90 substrates. Hsp90 may also act as a physiological ATP sensor by regulating the stability of growth-signaling cascades in relation to cellular energy charge, and network simulations reveal that molecular chaperones counterbalance perturbations in protein-folding dynamics that are associated with the sudden drops in ATP levels in diseased cells [25]. Therefore, the downregulation of these proteins suggests that 17-AAG may reduce cell proliferation, at least in part, by reducing ATP synthesis.

Some antiproliferative proteins, including GLUD1 (glutamate dehydrogenase 1), were upregulated and some proteins associated with the development and progression of tumors were downregulated upon exposure to 17-AAG. Glutamate activates the Ras/Raf/MEK/ERK cascade, and accelerates RPE cell proliferation [26]. Many proteins associated with cell structure and cell motility were also upregulated, including PLOD2 (procollagen-lysine, 2-oxoglutarate 5-dioxygenase 2), Moesin, Cofilin-1, FBXL17 (F-box and leucine-rich repeat protein 17) and CAPZA2 (F-actin capping protein subunit alpha-2). Some were downregulated, including STMN1 (Stathmin 1), LGALS1 (Galectin-1), PFN1 (Profilin-1) and COTL1 (Coactosin-like protein). Since molecular chaperones are involved differently but cooperatively in the formation and function of the eukaryotic cell cytoskeleton, inhibiting Hsp90 may change the expression of these proteins [27].

Proteins that are associated with ubiquitin-mediated proteasomal degradation mechanisms were differentially expressed [increased: PSMD14 (26S proteasome non-ATPase regulatory subunit 14), PSMC2 (26S protease regulatory subunit 7), PSMB2 (Proteasome subunit beta type-2); decreased: PSME1 (proteasome activator subunit 1 isoform 2), PSMB4 (Proteasome subunit beta type-4), PSMA1 (Isoform Short of Proteasome subunit alpha type-1)]. PSME1 not only activates the peptidase activity of 20 S proteasome, but also has a critical role as a cofactor which functions in concert with Hsc70 and Hsp40 during Hsp90-dependent protein refolding [28]. Interestingly, glyceraldehydes-3-phosphate dehydrogenase (GAPDH) was also downregulated, which was verified by the real-time PCR results obtained herein. And Hsp90 is reported to interact with GAPDH in HEK293 cells [29]. This protein is commonly used as an internal control in Western blots or real-time PCR. Although GAPDH changed slightly as the concentration of 17-AAG increased, GAPDH should not be used as the internal control in 17-AAG treatment research, especially in RPE cells.

Although several attempts were made herein to identify all of the proteins, there seven proteins whose changed by a factor of more than 1.5-fold were not identified. Neither Hsp90 nor Hsp70 was identified in this experiment, despite having been identified elsewhere [6,10]. Real-time PCR herein revealed that they were both upregulated after 16 h of treatment with 17-AAG. However, the expression level of Hsp90 is not correlated with inhibition by 17-AAG, as evidenced results obtained elsewhere [10,30]. 17-AAG inhibtis the protein HBP21 in ARPE-19 cells, which can interact with Hsp70 [31] and Hsp90 (unpublished results). Therefore, 17-AAG inhibited the function of Hsp90, but did not do so by changing the expression of Hsp90. Only a few Hsp90 client proteins, and no signaling proteins, were identified, probably because they were present in only small amounts [10]. Dongweon Song *et al*. [11] also found no alteration in the expression of signaling proteins upon exposure to a new Hsp90 inhibitor, IPI-504. Most of the proteins identified herein have no clear relationship with Hsp90, so 17-AAG may affect the Hsp90 client proteins that were not identified and thus indirectly affect the proteins that were identified herein.

Peroxiredoxin-1, Peroxiredoxin-2, Peroxiredoxin-3 and Peroxiredoxin-4, which are antioxidants, were also downregulated, while superoxide dismutase (SOD) was upregulated. SOD assay also reveals that SOD activity was upregulated in RPE cells upon exposure to 17-AAG for 16 h. These results suggest that 17-AAG may induce oxidative stress by inhibiting antioxidants. The Hsp90 inhibitors, geldanamycin and radicicol, both induce oxidative stress in cells [32-34]. Other researchers have found that each of the four Peroxiredoxins has a proliferative effect, and may be involved in the development or progression of cancer. Peroxiredoxin-4 also can activate NF-κB and c-Jun N-terminal kinase, and induces proliferation in fibroblasts [35,36], suggesting that the inhibition of the four proteins facilitates the antiproliferative effect of 17-AAG.

Proteins whose expression was identified as altered by 2-DE were analyzed further by IPA. Canonical pathway analysis indicated that oxidative stress was the top function in the list. Gene Ontology (GO) analysis revealed that the molecular function that contained the most identified proteins was oxidoreductase. Accordingly, Hsp70 and Hsp90α were greatly upregulated upon 16 h of treatment with 3 μM 17-AAG probably by oxidative stress. Finally, the proteomics results obtained using 17-AAG and other Hsp90 inhibitors were compared across various cell lines. The results for only a few proteins matched previous results [[6,10,25,37] and [38]], probably because of differences in the cell lines, treatment times and concentrations. Although IPA analysis reveals little in common among the proteins that were altered by other Hsp90 inhibitors, oxidative stress was placed highly in most function lists [[6,10,25,37] and [38]].

HSP90 has been reported to be strongly associated with oxidative stress. For example, Hsp90 protects against CYP2E1-dependent oxidant stress in HepG2 cells [26]. HSP90 inhibitors can induce oxidative stress in RPE cells and other cells, including radicicol and GA [34]. GA has a benzoquinone moiety that forms ROS (reactive oxygen species). Additionally, GA has been found to bind to GSH (glutathione), form stable GSH adducts and deplete cellular GSH. GA has been shown to generate superoxide. The applicatin of GSH has been reported to increase the resistance of cancer cells to benzoquinone ansamycin compounds [39]. Since 17-AAG is a derivative of the ansamycin geldanamycin and has a similar structure to that of GA, 17-AAG may also induce oxidative stress in many cell lines, including RPE cells.

## Conclusions

In conclusion, 17-AAG has a powerful antiproliferative effect on ARPE-19 cells by inducing cell apoptosis and cell cycle arrest. Although many other mechanisms pertain, low doses of 17-AAG may inhibit the proliferation of ARPE-19 cells in part by inducing oxidative stress in the early period of exposure. Although more experiments must be carried out to determine the precise role of oxidative stress in the mechanism of action of 17-AAG, 17-AAG was shown to reduce the proliferation of ARPE-19 cells and may be an excellent candidate for treating eye diseases associated with proliferative RPE cells, including PVR.

## Competing interests

The authors declare that they have no competing interests.

## Authors' contributions

JQY and QHL participated in the design of the study, performed major experiments, the data interpretation and drafted the manuscript. XC and QY participated in part of the experiments. ZYX performed part of proteomics analysis. FH and LW performed the flow cytometry and part of real-time PCR. JML designed the experiments and interpreted the data. All authors read and approved the final manuscript.

## Supplementary Material

Additional file 1 **Proteins upregulated in RPE cells following exposure to 17-AAG**. A table of name, IPI Accession number, function, molecular weight and isoelectric point of each protein upregulated in RPE cells following exposure to 17-AAG. Click here for file

Additional file 2 **Proteins downregulated in RPE cells following exposure to 17-AAG**. A table of name, IPI Accession number, function, molecular weight and isoelectric point of each protein downregulated in RPE cells following exposure to 17-AAG. Click here for file
