# Supplementary material for: Hsp90 inhibitor 17-allylamino-17-demethoxygeldanamycin inhibits the proliferation of ARPE-19 cells
Source: J Biomed Sci. 2010 Apr 23;17(1):30. doi: 10.1186/1423-0127-17-30 (PMC2873497; doi:10.1186/1423-0127-17-30)
Supplement: Additional file 1 — Proteins upregulated in RPE cells following exposure to 17-AAG. A table of name, IPI Accession number, function, molecular weight and isoelectric point of each protein upregulated in RPE cells following exposure to 17-AAG. [file 1423-0127-17-30-S1.DOC]

**Additional file 1. Proteins upregulated in RPE c**ells following exposure to 17-AAG.

| Spot ID | ENTRY NAME | Gene name | Full name | MW | PI | Score | Function |
| --- | --- | --- | --- | --- | --- | --- | --- |
| 567 | IPI00784154 | HSPD1 | 60 kDa heat shock protein, mitochondrial precursor | 61187 | 5.7 | 242 | Chaperone |
| 806 | IPI00013847 | UQCRC1 | Cytochrome b-c1 complex subunit 1, mitochondrial precursor | 53297 | 5.94 | 168 | protein binding/  ubiquinol-cytochrome-c reductase activity |
| 814 | IPI00337495 | PLOD2 | Isoform 2 of Procollagen-lysine,2-oxoglutarate 5-dioxygenase 2 precur | 87784 | 6.24 | 131 | Dioxygenase/  Oxidoreductase |
| 836 | IPI00021435 | PSMC2 | 26S protease regulatory subunit 7 | 49002 | 5.71 | 308 | ATPase activity/  protein binding |
| 926 | IPI00807545 | HNRNPK | Isoform 3 of Heterogeneous nuclear ribonucleoprotein K | 48760 | 5.38 | 151 | Ribonucleoprotein |
| 971 | IPI00465248 | ENO1 | Isoform alpha-enolase of Alpha-enolase | 47481 | 7.01 | 241 | Lyase/Repressor |
| 998 | IPI00027223 | IDH1 | Isocitrate dehydrogenase [NADP] cytoplasmic | 46915 | 6.53 | 87 | Oxidoreductase |
| 1049 | IPI00219365 | MSN | Moesin | 67892 | 6.08 | 127 | Structural protein |
|  | IPI00006558 | SH3GLB1 | Isoform 1 of SH3 domain GRB2-like protein B1 | 41056 | 5.78 | 66 | protein homodimerization activity |
| 1117 | IPI00017334 | PHB | Prohibitin | 29843 | 5.57 | 186 | protein binding/  transcription activator activity/transcription repressor activity |
| 1187 | IPI00024821 | PSMD14 | 26S proteasome non-ATPase regulatory subunit 14 | 34726 | 6.06 | 71 | protein binding |
|  | IPI00001539 | ACAA2 | 3-ketoacyl-CoA thiolase, mitochondrial | 42354 | 8.32 | 96 | Acyltransferase/  Transferase |
| 1221 | IPI00395332 | MCM9 | Uncharacterized protein MCM9 | 53696 | 8.26 | 62 | unknown |
| 1259 | IPI00873410 | CNN3 | 46 kDa protein | 45798 | 8.96 | 91 | actin binding/calmodulin binding/tropomyosin binding/troponin C binding |
| 1366 | IPI00011876 | MTAP | S-methyl-5'-thioadenosine phosphorylase | 31729 | 6.75 | 80 | Glycosyltransferase/  Transferase |
| 1369 | IPI00465028 | TPI1 | Isoform 1 of Triosephosphate isomerase | 31057 | 5.65 | 169 | Isomerase |
| 1448 | IPI00002520 | SHMT2 | Serine hydroxymethyltransferase, mitochondrial precursor | 56414 | 8.76 | 90 | Methyltransferase/  Transferase |
| 1493 | IPI00169383 | PGK1 | Phosphoglycerate kinase 1 | 44985 | 8.3 | 64 | Kinase/Transferase |
| 1499 | IPI00016801 | GLUD1 | Glutamate dehydrogenase 1, mitochondrial precursor | 61701 | 7.66 | 234 | Oxidoreductase |
| 1521 | IPI00479877 | ALDH9A1 | aldehyde dehydrogenase 9A1 | 57168 | 6.23 | 79 | Oxidoreductase |
| 1531 | IPI00007074 | YARS | Tyrosyl-tRNA synthetase, cytoplasmic | 59448 | 6.61 | 123 | Aminoacyl-tRNA synthetase  Ligase |
| 1540 | IPI00016801 | GLUD1 | Glutamate dehydrogenase 1, mitochondrial precursor | 61701 | 7.66 | 209 | Oxidoreductase |
|  | IPI00020906 | IMPA1 | Inositol monophosphatase | 30568 | 5.16 | 130 | Hydrolase |
| 1545 | IPI00022078 | NDRG1 | Protein NDRG1 | 43264 | 5.49 | 96 | protein binding |
|  | IPI00554788 | KRT18 | Keratin, type I cytoskeletal 18 |  |  |  | protein binding |
| 1611 | IPI00171844 | COPS4 | COP9 signalosome complex subunit 4 | 46525 | 5.57 | 225 | protein binding |
| 1732 | IPI00789755 | SEC13 | 34 kDa protein | 34504 | 5.4 | 85 | Protein transport/  Transport |
| 1794 | IPI00015018 | PPA1 | Inorganic pyrophosphatase | 33095 | 5.54 | 203 | Hydrolase |
| 1804 | IPI00217966 | LDHA | Isoform 1 of L-lactate dehydrogenase A chain | 36950 | 8.44 | 165 | Oxidoreductase |
| 1872 | IPI00026182 | CAPZA2 | F-actin capping protein subunit alpha-2 | 33157 | 5.57 | 178 | Actin capping |
| 1885 | IPI00220271 | AKR1A1 | Alcohol dehydrogenase | 36892 | 6.32 | 143 | Oxidoreductase |
| 1915 | IPI00301489 | UROD | Uroporphyrinogen decarboxylase | 41103 | 5.77 | 145 | Decarboxylase/Lyase |
| 2095 | IPI00019755 | GSTO1 | Glutathione transferase omega-1 | 27833 | 6.23 | 91 | Transferase |
| 2238 | IPI00003815 | ARHGDIA | Rho GDP-dissociation inhibitor 1 | 23250 | 5.02 | 128 | GTPase activation |
| 2240 | IPI00028006 | PSMB2 | Proteasome subunit beta type-2 | 22993 | 6.51 | 112 | Hydrolase/Protease/  Threonine protease |
| 2298 | IPI00375531 | NME2 | NME1 non-metastatic cells 1, protein (NM23A | 19869 | 5.42 | 104 | Activator/Anti-oncogene  /Kinase/Transferase |
| 2342 | IPI00012011 | CFL1 | Cofilin-1 | 18719 | 8.22 | 111 | protein binding |
| 2444 | IPI00411704 | EIF5A | Isoform 1 of Eukaryotic translation initiation factor 5A-1 | 17049 | 5.08 | 104 | Initiation factor |
| 2460 | IPI00218733 | SOD1 | Superoxide dismutase | 16154 | 5.7 | 93 | Antioxidant/  Oxidoreductase |
