# Supplementary material for: Hsp90 inhibitor 17-allylamino-17-demethoxygeldanamycin inhibits the proliferation of ARPE-19 cells
Source: J Biomed Sci. 2010 Apr 23;17(1):30. doi: 10.1186/1423-0127-17-30 (PMC2873497; doi:10.1186/1423-0127-17-30)
Supplement: Additional file 2 — Proteins downregulated in RPE cells following exposure to 17-AAG. A table of name, IPI Accession number, function, molecular weight and isoelectric point of each protein downregulated in RPE cells following exposure to 17-AAG. [file 1423-0127-17-30-S2.DOC]

**Additional file 2. Proteins downregulated in RPE c**ells following exposure to 17-AAG.

| Spot ID | ENTRY NAME | Gene name | Full name | MW | PI | Score | Function |
| --- | --- | --- | --- | --- | --- | --- | --- |
| 600 | IPI00025252 | PDIA3 | Protein disulfide-isomerase A3 precursor | 57146 | 5.98 | 256 | Isomerase |
| 609 | IPI00414320 | ANXA11 | Annexin A11 | 54697 | 7.53 | 233 | phospholipid binding/  protein binding |
| 951 | IPI00465248 | ENO1 | Isoform alpha-enolase of Alpha-enolase | 47481 | 7.01 | 171 | Lyase/Repressor |
| 1341 | IPI00795257 | GAPDH | glyceraldehyde-3-phosphate dehydrogenase 32 kDa protein | 31699 | 7.15 | 82 | Oxidoreductase |
| 1347 | IPI00411706 | ESD | S-formylglutathione hydrolase | 31956 | 6.54 | 124 | Hydrolase/  Serine esterase |
| 1422 | IPI00555956 | PSMB4 | Proteasome subunit beta type-4 precursor | 29242 | 5.72 | 81 | Hydrolase/Protease/  Threonine protease |
| 1443 | IPI00016832 | PSMA1 | Isoform Short of Proteasome subunit alpha type-1 | 29822 | 5.72 | 70 | Hydrolase/Protease/  Threonine protease |
| 1462 | IPI00873410 | CNN3 | 46 kDa protein | 45798 | 8.96 | 165 | actin binding/  calmodulin binding/  tropomyosin binding/  troponin C binding |
| 1492 | IPI00011229 | CTSD | Cathepsin D precursor | 45037 | 6.1 | 130 | Aspartyl protease/  Hydrolase/Protease |
| 1576 | IPI00147874 | NANS | Sialic acid synthase | 40738 | 6.29 | 111 | Transferase |
| 1608 | IPI00465439 | ALDOA | Fructose-bisphosphate aldolase A | 39851 | 8.3 | 163 | Lyase |
| 1618 | IPI00418262 | ALDOC | Fructose-bisphosphate aldolase C | 39830 | 6.41 | 143 | Lyase |
| 1626 | IPI00001539 | ACAA2 | 3-ketoacyl-CoA thiolase, mitochondrial | 42354 | 8.32 | 113 | Acyltransferase/  Transferase |
| 1628 | IPI00218918 | ANXA1 | Annexin A1 | 38918 | 6.57 | 288 | Phospholipase A2 inhibitor |
| 1687 | IPI00016610 | PCBP1 | Poly(rC)-binding protein 1 | 37987 | 6.66 | 124 | Ribonucleoprotein |
|  | IPI00418262 | ALDOC | Fructose-bisphosphate aldolase C | 39830 | 6.41 | 143 | Lyase |
| 1726 | IPI00549885 | PDHB | Isoform 2 of Pyruvate dehydrogenase E1 component subunit beta, mitocho | 37518 | 5.64 | 95 | Oxidoreductase |
| 1808 | IPI00217966 | LDHA | Isoform 1 of L-lactate dehydrogenase A chain | 36950 | 8.44 | 152 | Oxidoreductase |
| 1813 | IPI00219217 | LDHB | L-lactate dehydrogenase B chain | 36900 | 5.71 | 251 | Oxidoreductase |
| 1889 | IPI00872379 | ANXA5 | Uncharacterized protein ANXA5 (Fragment) | 35840 | 4.94 | 291 | phospholipase inhibitor activity/phospholipid binding/protein binding |
| 1890 | IPI00872780 | ANXA4 | Annexin A4 | 36088 | 5.84 | 225 | calcium ion binding/  calcium-dependent phospholipid |
| 1914 | IPI00848226 | GNB2L1 | Guanine nucleotide-binding protein subunit beta-2-like 1 | 35511 | 7.6 | 271 | receptor binding |
| 1930 | IPI00017334 | PHB | Prohibitin | 29843 | 5.57 | 208 | protein binding/  transcription activator activity/transcription repressor activity |
| 1958 | IPI00784190 | FBXL17 | F-box and leucine-rich repeat protein 17 | 34997 | 8.26 | 66 | ubiquitin-dependent protein catabolic |
| 1971 | IPI00748256 | PSME1 | proteasome activator subunit 1 isoform 2 | 28869 | 5.55 | 132 | proteasome activator activity /protein binding |
| 1977 | IPI00011937 | PRDX4 | Peroxiredoxin-4 | 30749 | 5.86 | 159 | Antioxidant/  Oxidoreductase  Peroxidase |
| 1992 | IPI00008530 | RPLP0 | 60S acidic ribosomal protein P0 | 34423 | 5.71 | 272 | Ribonucleoprotein/  Ribosomal protein |
| 2041 | IPI00795257 | GAPDH | 32 kDa protein | 31699 | 7.15 | 150 | Oxidoreductase |
| 2075 | IPI00220342 | DDAH1 | N(G),N(G)-dimethylarginine dimethylaminohydrolase 1 | 31444 | 5.53 | 141 | Hydrolase |
| 2088 | IPI00465028 | TPI1 | Isoform 1 of Triosephosphate isomerase | 31057 | 5.65 | 276 | Isomerase |
| 2090 | IPI00001960 | CLIC4 | Chloride intracellular channel protein 4 | 28982 | 5.45 | 190 | Chloride channel/  Ionic channel/  Voltage-gated channel |
| 2104 | IPI00029997 | PGLS | 6-phosphogluconolactonase | 27815 | 5.7 | 79 | Hydrolase |
| 2118 | IPI00010896 | CLIC1 | Chloride intracellular channel protein 1 | 27248 | 5.09 | 271 | Chloride channel/  Ionic channel/  Voltage-gated channel |
| 2180 | IPI00792352 | RAN | 26 kDa protein | 26678 | 8.51 | 221 | GTP binding |
| 2190 | IPI00374151 | PRDX3 | peroxiredoxin 3 isoform b | 26107 | 7.04 | 91 | Antioxidant/  Oxidoreductase/  Peroxidase |
| 2192 | IPI00026546 | PAFAH1B2 | Platelet-activating factor acetylhydrolase IB subunit beta | 25724 | 5.57 | 108 | Hydrolase |
| 2202 | IPI00026546 | PAFAH1B2 | Platelet-activating factor acetylhydrolase IB subunit beta | 25724 | 5.57 | 93 | Hydrolase |
| 2223 | IPI00027350 | PRDX2 | Peroxiredoxin-2 | 22049 | 5.66 | 140 | Antioxidant/  Oxidoreductase/  Peroxidase |
| 2232 | IPI00873680 | EIF4E | Uncharacterized protein EIF4E (Fragment) | 25310 | 5.63 | 100 | Initiation factor |
| 2259 | IPI00025512 | HSPB1 | Heat shock protein beta-1 | 22826 | 5.98 | 117 | identical protein binding |
| 2278 | IPI00025512 | HSPB1 | Heat shock protein beta-1 | 22826 | 5.98 | 149 | identical protein binding |
| 2293 | IPI00885126 | DNAH10 | DNAH10 variant protein | 382946 | 5.61 | 76 | Motor protein |
| 2312 | IPI00220766 | GLO1 | Lactoylglutathione lyase | 20992 | 5.12 | 105 | Lyase |
| 2330 | IPI00640741 | PRDX1 | Peroxiredoxin-1 19 kDa protein | 19135 | 6.41 | 149 | Antioxidant/  Oxidoreductase/  Peroxidase |
| 2470 | IPI00017704 | COTL1 | Coactosin-like protein | 16049 | 5.54 | 161 | actin binding/  enzyme binding |
| 2557 | IPI00216691 | PFN1 | Profilin-1 | 15216 | 8.44 | 168 | Actin-binding |
| 2570 | IPI00219219 | LGALS1 | Galectin-1 | 15048 | 5.34 | 147 | protein binding/  signal transducer activity |
| 2580 | IPI00643115 | STMN1 | Stathmin 1/oncoprotein 18 | 13597 | 9.76 | 67 | Developmental protein |
